# *In vivo* assessment of airway wall compliance during inhalation injury response using anatomical optical coherence elastography

**DOI:** 10.1117/1.JBO.30.7.076001

**Published:** 2025-07-03

**Authors:** Yinghan Xu, Srikamal Soundararajan, Scott H. Randell, Nicusor Iftimia, Gopi Maguluri, John Grimble, Carlton J. Zdanski, Amy L. Oldenburg

**Affiliations:** a University of North Carolina at Chapel Hill, Department of Biomedical Engineering, Chapel Hill, North Carolina, United States; b University of North Carolina at Chapel Hill, Biomedical Research Imaging Center, Chapel Hill, North Carolina, United States; c University of North Carolina at Chapel Hill, Marsico Lung Institute, Chapel Hill, North Carolina, United States; d Physical Sciences Inc., Andover, Massachusetts, United States; e University of North Carolina at Chapel Hill, Department of Otolaryngology/Head and Neck Surgery, Chapel Hill, North Carolina, United States; f University of North Carolina at Chapel Hill, Department of Physics and Astronomy, Chapel Hill, North Carolina, United States

**Keywords:** optical coherence elastography, airway compliance, inhalation injury

## Abstract

**Significance:**

Inhalation injury, a critical complication in patients with severe burns, contributes substantially to morbidity and mortality. Current diagnostic practices suffer from subjectivity and lack quantitative metrics. Enhanced diagnostic accuracy is imperative for improving treatment outcomes.

**Aim:**

Our objective was to develop normalized cross-sectional compliance (nCsC) of the airway wall, as measured by anatomical optical coherence tomography (aOCT), to reflect the severity of trachea inhalation injury.

**Approach:**

We employed a customized aOCT system that incorporates an intraluminal pressure probe to assess nCsC *in vivo* in pigs subjected to steam-induced inhalation injuries. Multiple steam intensity levels of injury were induced, and nCsC was measured from the carina to the larynx at time points up to 6 h using aOCT. Histological analysis was performed post-mortem.

**Results:**

We revealed that airway wall nCsC decreased initially after injury but exhibited recovery at 4 h. This is supported by ANOVA results showing that nCsC was significantly influenced by time (p=0.002). Linear regression indicated that nCsC was negatively correlated with anatomical position (p=0.047), whereas histological injury grade was positively correlated with position (p=0.015). In other words, nCsC decreased and injury grading increased when closer to the site of steam introduction.

**Conclusions:**

Airway wall nCsC is a promising quantitative metric for assessing inhalation injury. Future translation of this aOCT-based technology to humans may potentially enhance clinical management of inhalation injuries.

## 1 Introduction

Inhalation injury occurs from pulmonary exposure to a variety of chemicals, such as smoke, vapors, and gases.^1^ It constitutes a significant cause of morbidity and mortality in patients with severe burns.^2^ According to the Centers for Disease Control and Prevention, 398,000 burn injuries required medical treatment in 2021, resulting in 3800 deaths due to smoke inhalation.^3^ Inhalation injury affects the pulmonary system due to a combination of factors, including heat, hypoxemia, and the chemical effects of toxic inhalants.^4^ The high mortality rate associated with inhalation injury may be improved by standardized and objective diagnostic criteria. The current gold standard for diagnosing inhalation injury is flexible bronchoscopy and the use of the Abbreviated Injury Score criteria^5^ to determine injury severity. However, a drawback of the injury scoring system is its subjectivity, as interpretation may vary among examining physicians.^6^ Therefore, a more quantitative method is necessary to assess inhalation injury effectively and inform evolving strategies for burn treatment. Here, we address this need by exploring a method for endoscopic, optical coherence elastography that has the potential to quantitatively map the heterogeneous response of the airway to injury via its reduced wall compliance.

Several imaging modalities have been previously explored for quantitative diagnosis of inhalation injury, including chest radiographs (X-rays),^7^ nuclear medicine,^8^ and chest computed tomography.^9^ Although these methods can offer diagnostic value, their limited sensitivity, resolution, and ionizing radiation hamper their utility for following the progression of inhalation injury. Ultrasound (US) has been widely used for point-of-care injury assessment and has successfully monitored upper airway wall thickness after smoke injury over the span of 2 days.^10^ However, US lacks the displacement resolution needed to quantify airway wall deformability (compliance) during respiratory pressure cycling, which is a key biomarker of injury. Researchers have demonstrated that loss of lung compliance and increase in airway resistance are related to the severity of inhalation injury through lung function measurements.^11^ However, lung function measurements are global and do not provide a spatially resolved relationship between wall compliance and underlying epithelial damage. Notably, one study showed that vocal fold stiffness increased at 3 days after burn injury compared with no injury, then decreased again after 7 days; measurements were acquired by using an indentation method.^12^ As such, an elastography method is desired to better understand the extent and severity of inhalation injury, as well as to potentially monitor its progression or recovery. Magnetic resonance elastography^13^ and endobronchial ultrasound elastography^14^ can be used to diagnose respiratory diseases; however, their resolution is insufficient to capture respiratory-induced airway deformation.

Optical coherence tomography (OCT) offers micron-scale resolution and may be employed endoscopically via fiber optics, facilitating visualization of luminal tissue and organs. Leveraging the benefits of traditional OCT, endoscopic OCT has been employed in the study of airway structure, such as smooth muscle^15^ and cilia.^16^ Endoscopic OCT has been utilized in *ex vivo*^17^ and *in vivo* studies^17^^–^^20^ involving small animals such as rabbits^17^ and larger animals such as sheep^18^ and pigs^19^ to assess changes in airway wall thickness after inhalation injury. Specifically, the rabbit model revealed that the normalized thickness of the mucosal layer increased after inhalation injury.^17^ Chemical inhalation injury can also lead to the thickening of the mucosal layer, where there was a significant correlation between the mucosal thickness and peak inspiratory pressure.^19^ A recent study investigating chemical inhalation injuries in a rabbit model found that the volume of the rabbit tracheal wall initially increased before subsequently decreasing.^21^ Anatomic OCT (aOCT) is an extension of endoscopic OCT that offers a longer imaging range needed for capturing the upper and central airway lumen.^22^ Importantly, aOCT measurements of the airway lumen’s deformation, combined with pressure measurements during breathing, can be used to estimate the compliance of the airway wall *in vivo*. Williamson et al.^23^ used aOCT and transpulmonary pressure measurements in the central airways of patients with obstructive lung diseases to assess airway compliance (defined as change in luminal area per unit pressure, which we call cross-sectional compliance or CsC in this study) and specific compliance [obtained by dividing CsC by luminal area, which we call normalized cross-sectional compliance (nCsC) in this study, to control for varying airway size and better reflect the intrinsic tissue stiffness, as discussed below]. Our group has recently deployed an intraluminal pressure probe with an aOCT imaging probe to more accurately measure pressure along the airway and better estimate CsC.^24^ The system was subsequently used to quantify CsC in *ex vivo* pig tracheae under heat injury conditions,^25^ revealing a negative correlation between aOCT elastography-derived compliance and the severity of heat injury. Subsequently, Bu et al.^26^ utilized a localized compliance (LC) metric to assess the mechanical properties of *ex vivo* porcine tracheas and found that the LC patterns corresponded with histological identification of stiff cartilage and soft muscle regions.

In this study, we chose to focus on normalized cross-sectional compliance (nCsC) as a simplified metric that reflects the underlying airway wall mechanical properties and induces different steam intensity levels via steam treatment of *in vivo* pig airways. We hypothesize that aOCT-derived nCsC decreases initially in response to injury, with a potential to reveal long-term recovery via gradual subsequent increase. In addition, we hypothesize that nCsC reflects the steam intensity level of the injury, with increased intensity level (and corresponding increased histologic injury severity grading) correlating with reduced nCsC along the length of the trachea. To test these hypotheses, we performed aOCT imaging with simultaneous intraluminal pressure measurement up to 6 h post-injury, collecting pullback scans from the carina to the larynx. Collected data were used to compute nCsC and associate the data as a function of time post-injury, steam intensity level, and anatomical position. Airway tissues were also sampled at the end of each procedure for histological analysis of injury severity, and injury gradings by histology were similarly associated with steam intensity level and anatomical location along the trachea.

## 2 Materials and Methods

### 2.1 Imaging and Ventilation Systems

**Fig. 1:**
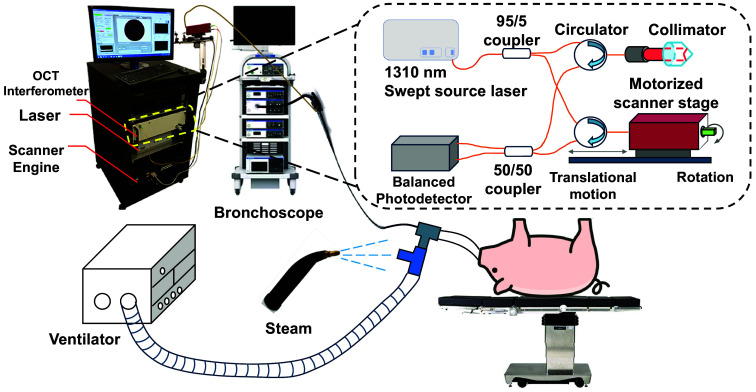
aOCT system and experimental set up for in vivo steam injury. The aOCT catheter and pressure catheter are introduced via the bronchoscope working channel.

To capture airway changes before and after inhalation injury and to study airway compliance, we used an endoscopic aOCT system, designed and fabricated by Physical Sciences Inc., Andover, Massachusetts, United States, integrated with a pressure sensing system similar to a system previously reported by our research group.^27^ The aOCT system features a vertical-cavity surface-emitting laser wavelength-swept source (SL1310V1, Thorlabs Inc., Newton, New Jersey, United States) with a center wavelength of ∼1310  nm, a sweep range of 95 nm, and a sweep rate of 200 kHz. Light passes through a Mach–Zehnder interferometer with a variable reference arm. The produced interference signal is captured by a balanced photodetector (PDB480C, Thorlabs Inc., Newton, New Jersey, United States) and digitized using a data acquisition (DAQ) card (ATS9360, Alazar Technologies Inc., Pointe-Claire, Canada), as shown in Fig. 1. A custom, ball lens, fiber-optic catheter (Physical Sciences Inc. Andover, Massachusetts, United States) enclosed in a sheath with an outer diameter of 0.85 mm is scanned via a rotational/translational stage (Physical Sciences Inc., Andover, Massachusetts, United States) to provide imaging of the airway lumen; in this study, only the rotational stage was used while varying positions along the airway were assessed by repositioning the catheter tip among each scan. The system’s measured axial resolution is 10  μm with a lateral resolution of 35  μm at the focal position. The aOCT system sensitivity is 105 dB at 1 mm from the catheter tip, decreasing to 55 dB at 8.5 mm with 12.5 mm imaging range.

aOCT scans were collected with a rotation rate of 40 Hz. The total number of frames per scan (where a frame is defined as one rotation) is set to ∼500 to ensure at least three full breath cycles are captured with a total scanning time of ∼12.5  s. aOCT data were processed and displayed in real-time using GPU-accelerated software developed by Physical Sciences Inc., Andover, Massachusetts, United States. An intraluminal pressure sensor was integrated into the aOCT system. The pressure catheter is 1 mm in outer diameter (SPR-330A, Millar Inc., Pearland, Texas, United States) with a sensitivity of ∼1.32  cm $H_{2}O$ at an acquisition rate of 5 kHz through the pressure DAQ card. Both the aOCT catheter and the pressure catheter were introduced into the airway through an endoscope with a working channel of 2 mm (EVIS X1, Olympus America Inc., Bartlett, Tennessee, United States). The aOCT system is controlled through LabVIEW (National Instruments, Austin, Texas, United States), and the OCT image and pressure measurement are displayed in real time on the LabVIEW front panel. A SERVO 900C ventilator (Siemens-Elema AB, Solna, Sweden) was connected to the endotracheal tube (ETT) through a double swivel adapter to ventilate the animal. The ventilator is set to pressure-controlled ventilation mode with a respiratory rate of 20  breaths/min and a maximum inspiratory pressure (MIP) of 18 cm $H_{2}O$.

OCT catheter positioning within the airway was performed under endoscopic guidance. A dual-channel [white light (WL) and narrowband imaging (NBI)] endoscope (EVIS X1, Olympus America Inc., Bartlett, Tennessee, United States) was used. NBI is an endoscopic technique employing narrowband spectral optical filters to enhance the visualization of epithelial and subepithelial microvascular patterns.^28^ Superficial mucosal lesions typically missed by standard WL endoscopy are often more effectively identified in NBI due to their neoangiogenic patterns.^28^ The endoscope can switch between WL and NBI light using a button control. Both the aOCT catheter and the pressure catheter were introduced into the airway through the endoscope, allowing visualization of the catheters’ positions. In each scan, the physician first positioned the OCT catheter tip to protrude from the bronchoscope channel so that the laser beam exiting the catheter was visible and then placed the pressure catheter tip slightly proximal to it—just behind the OCT laser tip—while keeping both catheters beyond the tip of the bronchoscope channel. Although the exact separation may have varied slightly, we consistently aimed to position the pressure catheter as close as possible to the OCT scanning site without interfering with image acquisition.

### 2.2 Animal

Three young pigs were anesthetized and intubated with cuffed ETTs of size 5.0 with an inner diameter of 5 mm before imaging. The pigs were then connected to the ventilator through a double swivel adapter and a 3-way connector and then placed in a supine position on a stable platform. All experimental procedures were approved by the Institutional Animal Care and Use Committee at the University of North Carolina, Chapel Hill.

A commercial steam cleaner (I500b, Reliable Corp, Canada) was used to induce heat injury by introducing steam into each pig’s airway with an operating pressure of 50 psi at 105°C. The steam blasts were manually controlled by the physician, with each blast initiated by pushing the handle and ending when it reached the fully depressed position and delivered continuously, with each blast immediately following the previous one, resulting in a consistent duration across blasts. Three different steam intensities were used (one for each pig) to induce different steam injury levels: low intensity (5 blasts of steam), medium intensity (10 blasts of steam), and high intensity (20 blasts of steam). For the low steam intensity, multiple aOCT airway scans were performed ∼1  cm apart from the distal end around the carina to the proximal end around the larynx. For the medium and high steam intensities, four positions were chosen for aOCT scans that sequentially moved proximally in the airway in nearly equidistant increments: the distal end of the trachea near the carina, a second position distal to the middle of the trachea, a third position proximal to the middle of the trachea, and the proximal end near the larynx. The same aOCT scan procedures were followed for all time points: before treatment (low, medium, and high steam intensity), immediately after injury (low, medium, and high steam intensity), 30 min post-injury (low steam intensity), 1 h (low, medium, and high steam intensity), 2 h (low, medium, and high steam intensity), 4 h (low and medium steam intensity), and 6 h (low steam intensity). WL and NBI images were also collected after the catheters were in position for each aOCT scan (but before each corresponding aOCT scan).

Pigs with low, medium, and high steam intensity were euthanized 6, 4, and 2 h after the injury, respectively, following the completion of scanning. The tracheae with attached larynges were formalin-fixed, sliced into ∼1  cm segments, paraffin-embedded, sectioned into 4  μm slices, stained with hematoxylin and eosin (H&E), and slide mounted. Each slide was coded and randomized before being given to an observer for blinded grading. The observer, with over 40 years of experience in respiratory tract experimental pathology and histopathology, graded the slides from 1 to 3, with 1 indicating low injury, exhibiting normal epithelium and lamina propria, 2 indicating moderate injury, exhibiting mild-to-moderate epithelial and lamina propria abnormalities including cell necrosis, edema, or inflammation, and 3 indicating severe injury, exhibiting severe epithelial and lamina propria abnormalities, including epithelial sloughing, hemorrhage, severe inflammation.

### 2.3 Data Processing

The aOCT images were computed from raw binary data using a digital dispersion compensation algorithm based on entropy minimization in MATLAB.^29^ The computed aOCT images were averaged in groups of four A-lines, culminating in image frames of 1152 pixels in depth by 1248 pixels around the circumference, where the depth represents 12.5 mm in physical distance. Then, the air–tissue interface of the airway lumen in each frame was segmented via a custom semi-automatic segmentation method validated previously.^30^ In some aOCT images, the airway extended beyond the 12.5 mm imaging range from the catheter tip; in these images, the airway wall was still apparent via an aliasing artifact, and a manual correction method was employed to obtain the correct airway geometry. Figure S1 in the Supplementary Material illustrates the manual correction of segmentation errors in the aliasing region, where the surface that appears lower is selected in preference to the upper surface. Subsequently, the column indices where the segmentation line reaches the bottom of the image are documented. These indices are used to mirror the aliasing region beyond the 12.5 mm image depth (correcting for how aliasing “flips” the image of objects beyond the working distance). The original segmentation image is then updated to integrate the inverted aliasing region.

The segmented airway lumen was then used to calculate the cross-sectional area (CSA) of the airway for each aOCT frame. The mean and standard deviation of the 5 kHz pressure data were computed within the duration of each 40 Hz aOCT frame to generate temporally synchronized pressure—CSA data pairs for each aOCT scan. Before each scan, we attempted to adjust the length of the reference arm to match the sample arm, the latter of which varies with the fiber-optic catheter length. However, we found that there were still variations up to 1.2 mm in the collected data. To mitigate this variation, we used the outer sheath diameter (in pixels) from a rigid tube phantom with a known physical diameter as a reference standard. All sheath sizes in the dataset were then corrected relative to this reference, ensuring consistent outer sheath dimensions for accurate airway cross-sectional area calculations. At least two breath cycles were used to determine the maximum and minimum CSA and pressure, respectively, in each cycle. Then, the average minimum and maximum CSAs and pressures across the breath cycles were, respectively, calculated, and the changes in CSA (∂CSA) and pressure (∂p) were calculated by subtracting the respective averaged minima from the averaged maxima. Similarly, the time-averaged CSA, $\overset{¯}{\mathrm{CSA}}$, was calculated by averaging all pairs of maxima and minima. The standard deviation of the CSA was calculated based on the changes in CSA over the five frames preceding and following the frames with the maximum and minimum CSA values. The standard deviation of the averaged CSA is the root sum of the maximum and minimum CSA standard deviation.

From the above measurements, it is possible to estimate the airway wall compliance under certain simplifying assumptions, including that the airway wall is elastically homogeneous and isotropic, the loads from pressure are symmetrically distributed around the airway’s axis, and the mechanical response is linearly elastic. Using these simplifications, the cross-sectional compliance (CC) may be defined as the derivative of CSA with respect to pressure (p)

$$CC=\frac{\partial \mathrm{CSA}}{\partial p},$$ (1)

which is computed for each aOCT scan. nCsC is subsequently defined as CC divided by the average cross-sectional area ($\overset{¯}{\mathrm{CSA}}$)

$$\mathrm{nCsC}=\frac{\mathrm{CC}}{\overset{¯}{\mathrm{CSA}}}.$$ (2)

Normalization removes the effect of size or scale differences among samples. nCsC can then be reported as a percent area change per unit pressure, via

$$\mathrm{nCsC}=\frac{\partial \mathrm{CSA}/\overset{¯}{\mathrm{CSA}}}{\partial p}*100\%.$$ (3)

If we consider a thick-walled deformable tube model, compliance depends on the material properties (e.g., Young’s modulus) and the geometry of the tube (e.g., inner and outer radii). Although the cross-sectional compliance depends on geometric dimensions, the normalization process makes the $\partial \mathrm{CSA}/\overset{¯}{\mathrm{CSA}}$ term dimensionless, thus making nCsC independent of the absolute size or scale of the samples (as shown in the Supplementary Material). This allows for a more direct comparison of airway wall mechanical properties across samples with varying cross-sectional areas.

The collected nCsC data were analyzed using R Studio (RStudio Team, PBC, Boston, Massachusetts, United States, 2020). Two multiway ANOVA were conducted using independent variables of steam intensity level, time (in hours), and anatomical location, where the dependent variable was nCsC. Numerical values were assigned to steam intensity level as follows: low intensity = 1, medium intensity = 2, and high intensity = 3. Numerical values for anatomical positions were assigned from 0 to 1, where the distal end of the airway near the carina was labeled position 0, and the proximal end of the airway near the larynx was labeled 1. Numerical values for time were assigned as follows: before steam treatment = −1, after steam treatment = 0, 30 min = 0.5, and subsequent time points were assigned values corresponding to their hour marks. All intermediate positions were labeled according to their relative positions in this distance system.

Interaction terms were also explored in ANOVA for the independent variables by multiplying two or more factors: these included the products of steam intensity level and time, steam intensity level and position, time and position, and a comprehensive interaction term involving the product of steam intensity level, time, and position. ANOVA was also employed to assess the histologic injury grading for injury severity in relation to steam intensity level and position with the injury grading being the dependent variable and steam intensity level and position being the independent variables.

## 3 Results and Discussion

### 3.1 aOCT Imaging and Extraction of CSA and Pressure

Following injury, a marked increase in airway secretions was observed. Immediately after the injury was induced, the superficial tissue exhibited a pale, cooked-like appearance in white light imaging under the bronchoscope. Medium and high steam intensity levels were characterized by bleeding and visibly enlarged vasculature when examined with white light bronchoscopy. There were challenges associated with maintaining the aOCT catheter in a position that avoided contact with the airway wall, due to the pigs’ breathing movements, which was necessary for optimal imaging. In addition, the increased secretion of mucus and fluids made it more likely they would adhere to the aOCT catheter. In the case of high steam intensity level, the pig succumbed 10 min after steam injury induction. Observations indicated that the high steam intensity level airway contracted and failed to maintain its normal shape, in contrast to what was seen with low and medium steam intensity levels.

**Fig. 2:**
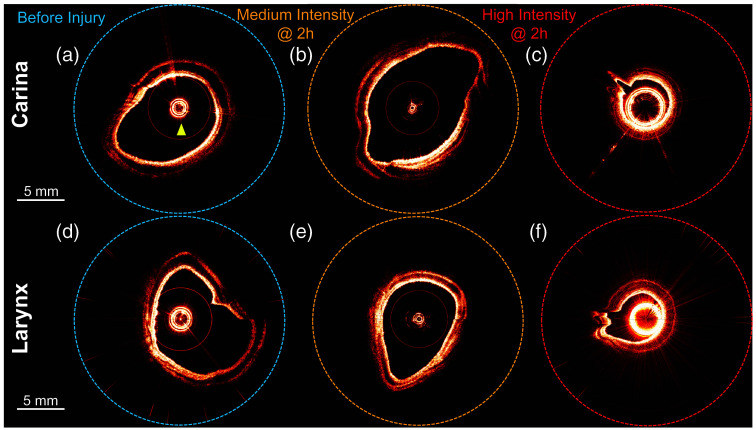
Representative aOCT images of pig airways. (a)–(c) Display cross-sections immediately above the carina, and (d)–(f) display cross-sections near the larynx. (a) High steam intensity level pig before treatment. Yellow arrow indicates the OCT catheter. (b) Medium steam intensity level pig 2 h post-injury. (c) High steam intensity level pig 2 h post-injury. (d) High steam intensity level pig before treatment. (e) Medium steam intensity level pig 2 h post-injury. (f) High steam intensity level pig 2 h post-injury. The apparent difference in catheter sheath size across the images is due to variations in the reference arm length of the OCT system.

Figure 2 shows representative aOCT images of the pig airways from two positions: proximal to the carina (distal end) and proximal to the larynx (proximal end). Representative data showing how the aOCT lumen was segmented for compliance analysis (displayed as a green line overlaid on the OCT images) are provided in Video 1. Due to the challenges noted above, aOCT scans at certain positions and times across all three steam intensity levels were found to be unsuitable for compliance analysis. The aOCT scans selected for elastography all exhibit a clear boundary between the tissue and the sheath of the aOCT catheter. In the low steam intensity level experiment, pre-injury aOCT images displayed aliasing artifacts due to the lumen extending beyond the imaging radius; these were still able to be analyzed using the custom method described above. Due to limited resources, no uninjured pigs were used as controls in this experiment. However, the “before treatment” time point effectively served as a control for each airway [Figs. 2(a) and 2(d)] and also had the advantage of reducing pig-to-pig variance and providing a more reliable baseline. After 4 h, the airways with low and medium steam intensity levels maintained their pre-injury luminal shape [Figs. 2(b) and 2(e)]. However, the airway lumen with severe steam intensity level displayed a reduced CSA 1 h post-injury compared with its pre-injury state [Figs. 2(c) and 2(f)]. Notably, the pig with a high steam intensity level succumbed 10 min post-treatment, and data were collected 1 h post-treatment.

**Fig. 3:**
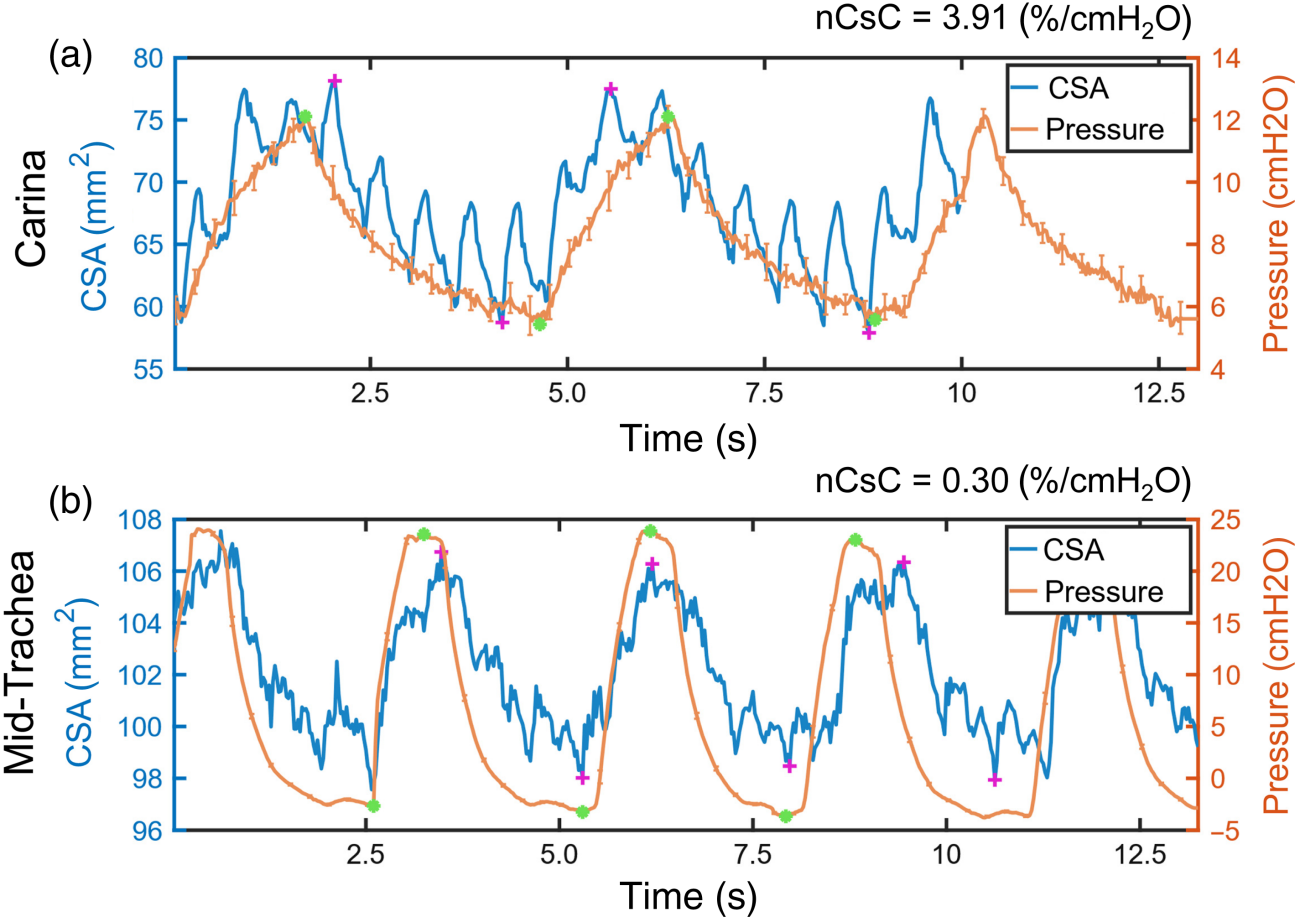
Example datasets of CSA and intraluminal pressure versus time. (a) Airway cross-section immediately above the carina in the low steam intensity level pig before treatment. (b) Airway cross-section near the larynx in the medium steam intensity level pig 4 h post-injury. The maximum and minimum CSA and pressure used to calculate nCsC in each cycle are marked with purple crosses (CSA) and green dots (pressure).

CSA and pressure measurements were extracted from the available aOCT datasets. Figure 3 shows CSA and pressure over time for the scans exhibiting the highest and lowest nCsC values; the highest nCsC was obtained before treatment at the carina for the low steam intensity level pig [Fig. 3(a)], and the lowest nCsC was obtained 4 h post-injury at the mid-trachea for the medium steam intensity level pig. Although CSA and pressure were generally well correlated in time during respiration, hardware limitations caused asynchronization between CSA and pressure measurements; this issue was mitigated by computing nCsC from only the minimum and maximum CSA and pressure values for each respiratory cycle. The average standard deviation of minimum CSA, maximum CSA, and mean CSA for all aOCT scans processed is 0.97, 0.71, and $1.3\;{\mathrm{mm}}^{2}$, respectively. These standard deviations in CSA are small compared with the variation observed during respiration (Fig. 3), indicating that aOCT offers sufficient resolution to estimate wall compliance from respiratory-induced lumen deformation. It is interesting to note that the pressure curves varied at different positions along the length of the airway: pressure measurements near the proximal end (larynx) exhibited a lower modulation amplitude (max–min) compared with those near the distal end (carina) (Fig. S2 in the Supplementary Material). This may be attributed to the pressure at the proximal end being more affected by the ventilator, leading to a smaller pressure modulation amplitude. It is also worth noting that the additional modulation at higher frequencies in CSA, as shown in Fig. 3(b), may be correlated with the cardiac oscillation.

### 3.2 aOCT Elastography

**Fig. 4:**
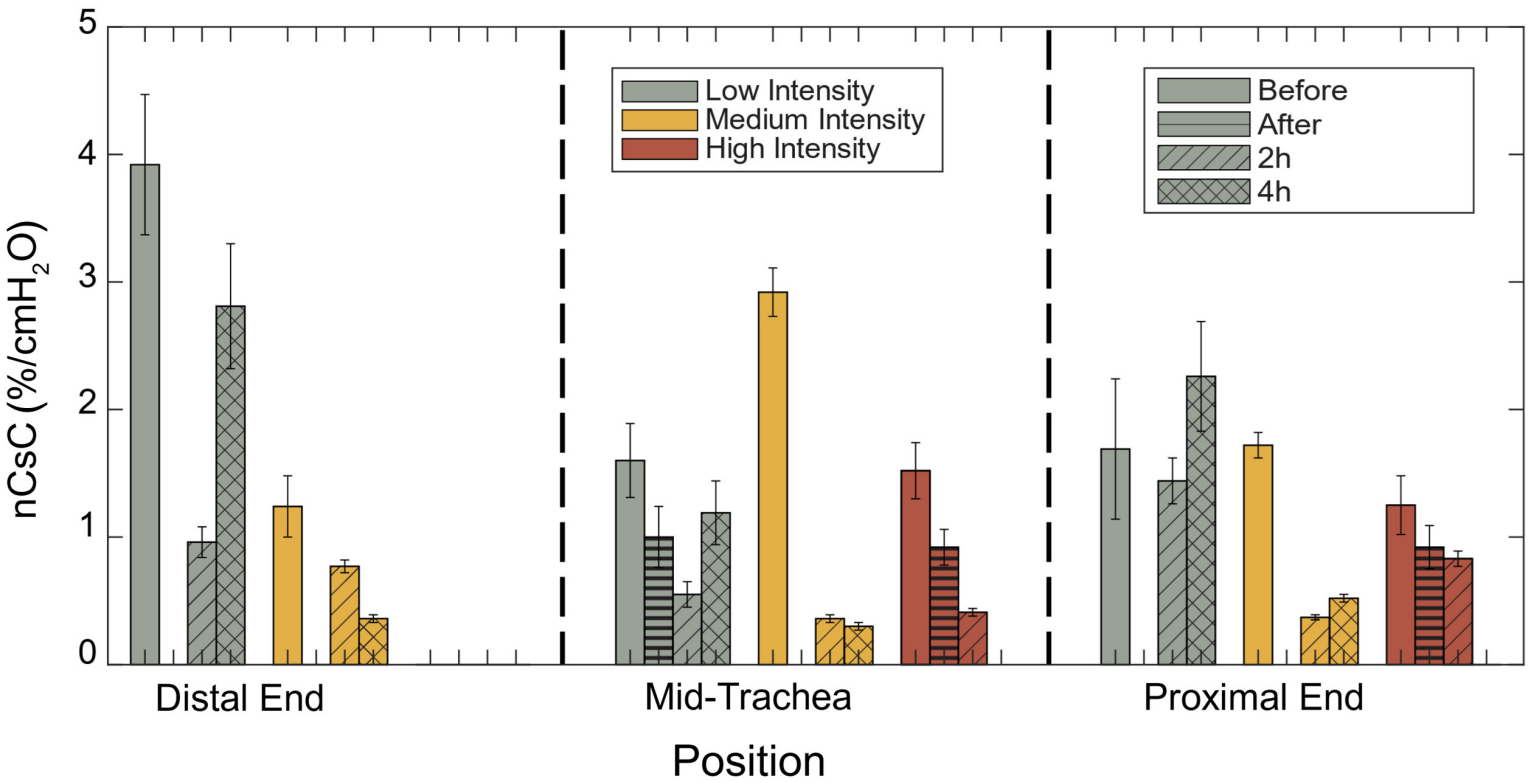
Normalized cross-sectional compliance (nCsC) with respect to steam intensity level, time post-injury, and position along the airway. The high-intensity measurements at 2 h were obtained from a post-mortem subject.

nCsC were calculated from the CSA and pressure measurements according to Eqs. (1)–(3). A complete table of average CSA, CC, and nCsC for each aOCT scan can be found in Table S1 in the Supplementary Material. Figure 4 displays a summary of nCsC in relation to steam intensity level, time post-injury, and position along the airway. The data in Fig. 4 are sorted by four time points, before treatment, immediately after injury, 2 h post-injury, and 4 h post-injury, and are also binned into three sections of the trachea: the distal end near the carina, the mid-trachea, and the proximal end near the larynx.

As shown in Fig. 4, nCsC decreases immediately and 2 h after the injury compared with before, for all data except the medium steam intensity level pig at the distal end. At 4 h, a recovery trend (increase in nCsC relative to 2 h data point) was observed in all available data except for the medium steam intensity level pig at the distal end. The first observation supports our hypothesis that nCsC initially decreases in response to steam injuries, which was formulated based on prior research of airway inhalation injury showing a correlation among loss of compliance (or increase of stiffness) in the airway in response to injury.^11^^,^^12^^,^^25^ Increased tissue stiffness is expected from a burn, which was observed in the white light bronchoscopy images, and edema (swelling), which is known to occur post inhalation injury.^21^

For the low steam intensity level pig, nCsC shows recovery after 4 h across all positions, highlighting the utility of aOCT elastography in monitoring injury progression and recovery. The compliance recovery over a prolonged post-injury period aligns with findings by Malka et al.,^12^ who reported vocal fold stiffness recovery from 3 to 7 days post-injury, and Zhu et al.,^21^ where the rabbit tracheal wall volume increased initially and decreased subsequently. ANOVA results (Table S2 in the Supplementary Material) indicate statistical significance for the time factor (p=0.002), suggesting a significant change in nCsC with time to support the time-dependent trends observed in Fig. 4. Furthermore, the interaction between steam intensity level and anatomical position may have an effect on nCsC, (p=0.076). Linear regression results (Table S3 in the Supplementary Material) suggest that there is a negative correlation between nCsC and both intensity level and position, the latter indicating that nCsC decreases when a position is closer to the proximal end, where the steam was introduced. Together, these results suggest that a high steam intensity level, particularly near the steam introduction site, reduces nCsC, supporting our second hypothesis.

Here, we chose to compute nCsC, which is CC normalized by the average CSA, to reduce the impact of airway lumen size on the CC. This choice is especially notable for the pig with a high steam intensity level, where the airway contracted in response to injury; given a constant CC, airway contraction in itself would cause nCsC to increase, yet, the result of injury response in this experiment was for nCsC to decrease (because CC decreased more than the CSA decreased). This provides a strong argument for the underlying elastic property of the airway wall being modified by the injury response.

As noted above, modeling the airway wall mechanical properties by a simplified nCsC metric involves several simplifying assumptions. One necessary assumption is that the airway wall is elastically homogeneous within a given cross-section. Although this neglects the inherently C-shaped structure of the pig’s cartilage, such inhomogeneities can be observed with aOCT by an LC metric.^26^ Another assumption is that the airway wall is linearly elastic, allowing us to employ a linear model and disregard any time lag between pressure changes and CSA alterations (the latter of which may be due to viscoelasticity or hardware asynchronization). In prior work,^24^ we showed that CSA and pressure in *ex vivo* pig tracheas were highly linearly related, supporting the validity of this assumption, although another study^27^ indicated there may be a significant time lag between pressure onset and CSA deformation during pig respiration.

### 3.3 Morphologic and Histologic Analyses

**Fig. 5:**
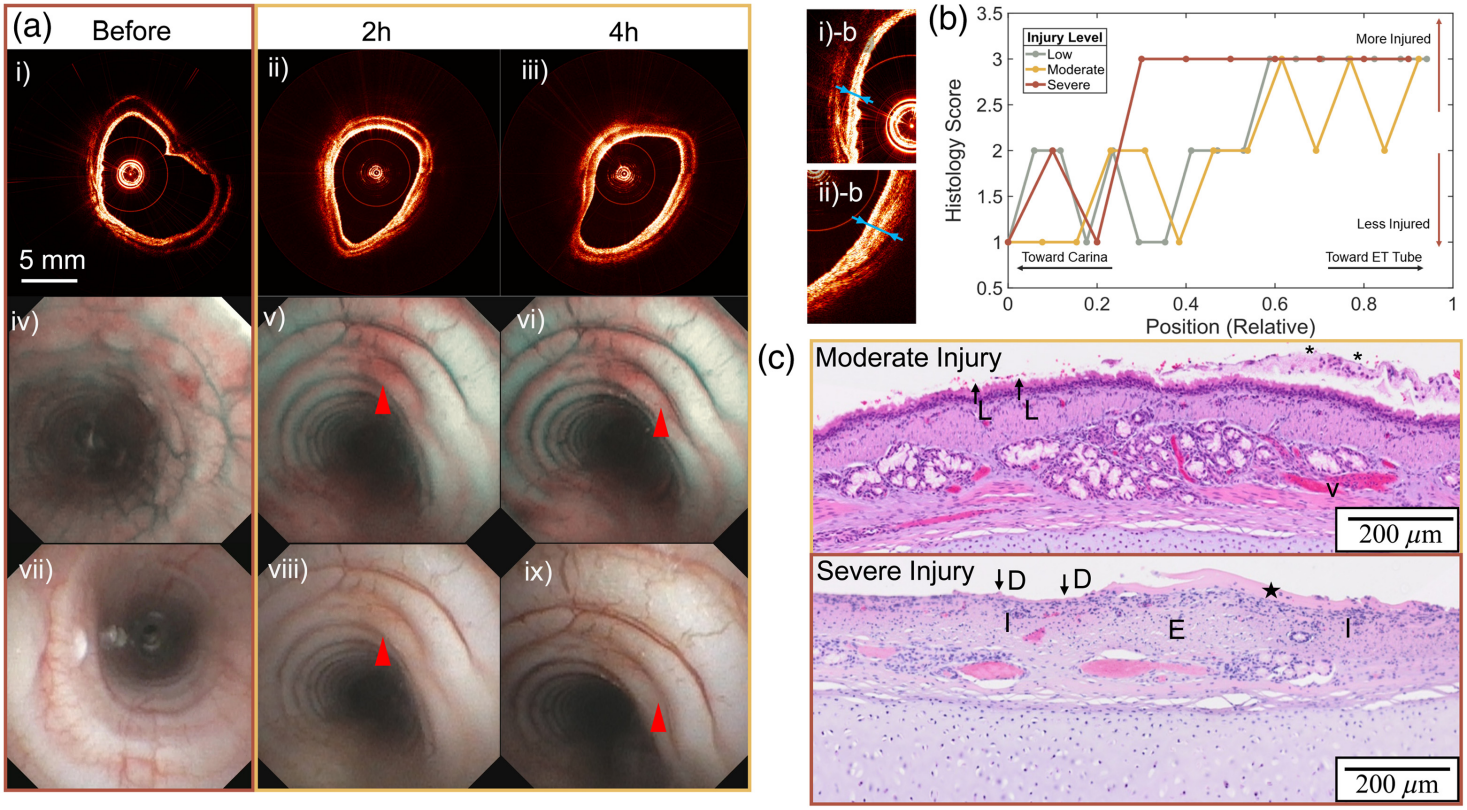
(a) (i–iii) aOCT images of a pig larynx before and at 2 and 4 h after injury. The before-treatment aOCT image is from the pig later induced with severe steam intensity, and 2 and 4 h aOCT images are from that with medium steam intensity. (iv–vi) Narrowband images at the larynx before and at 2 and 4 h after medium steam injury. Red arrows indicate blood vessel differences not observed in corresponding white light images in (vii–ix). (vii–ix) Corresponding white light images of the proximal trachea. (i)-b and (ii)-b: Zoomed region from (i) and (ii), blue arrows showing the epithelial cells with apparent thickening after the injury. (b) Histologic injury grading with respect to the relative position in different injury severities: 1 is low, 2 is moderate, and 3 is severe. Position 0 corresponds to the distal end near the carina, and 1 corresponds to the proximal end near the larynx. (c) Examples of H&E histology images of a moderate and severe injury that were graded 2 and 3, respectively, by the observer both at position 0.3. L–loss of cilia/epithelial disorganization. Asterisks—sloughed epithelial and mucus, inflammatory cells. V—vascular congestion. D—epithelial denudation. I—inflammation. E—edema. Star—proteinaceous membrane formation, inflammation.

Figure 5(a) presents a series of aOCT images [Fig. 5(ai)–5(aiii)] alongside WL images [Fig. 5(aiv–avi)] and NBI images [Fig. 5(avii)–5(aix)]. NBI provides enhanced details about tissue injury compared with WL images, as highlighted by the red arrow in Fig. 5(a). Although NBI reveals blood diffusion through the epithelial surface not visible in WL images, it fails to distinguish changes between 2 and 4 h post-injury. By contrast, aOCT images directly visualize changes in airway wall structure over time. For instance, aOCT images from before the injury and 2 h post-injury [Figs. 5(i-b) and 5(ii-b)] qualitatively show the thickening of the epithelial cell layer, corroborating findings by Yin et al.^17^ Moreover, nCsC offers a quantitative method to differentiate the severity of steam injury intensity at various time points.

After euthanasia, the pigs’ tracheae were extracted for histological analysis. Each histology slide was evaluated by an observer. For the histology assessment, each slide was coded and randomized before evaluation. The observer—blinded to both the anatomical location and steam intensity—graded the injury severity based solely on the presence of pathological features. The purpose of the histology scoring was to quantify the extent to which steam treatment elicited a burn response in the tissue at each sampled location, not necessarily to directly track the input steam levels. It is important to note that the histology slices were collected at the end for each subject due to logistical constraints and the invasive nature of tissue harvesting. By contrast, OCT imaging was performed at multiple time points specifically to evaluate the hypothesis that tissue recovers over time. This allowed us to monitor dynamic changes in tissue structure longitudinally without additional invasive procedures. In addition, the quality of histology slides is heavily influenced by the airway extraction process and handling during tissue fixation, which can subsequently impact the histologic grading. Figure 5(c) shows representative histology slides of tissue that appeared moderately injured and were graded “2” (which was from the medium steam intensity level pig) and tissue that appeared severely injured with a grading of “3” (from the high steam intensity level pig). The moderately injured tissue exhibits mild-to-moderate abnormalities in the epithelial and lamina propria layers, including loss of cilia, epithelial disorganization, sloughed epithelial and mucus, inflammation, and vascular congestion. By contrast, the severely injured tissue displays more pronounced changes, such as epithelial denudation, proteinaceous membrane formation, inflammation, and edema. This emphasizes the stark differences in tissue damage between moderate and severe injuries. It is noted that the impact of underlying pathological changes in the airway wall tissue, such as edema and necrosis, on the mechanical properties of the airway remains unknown. Heat injury is a complex process. Rong et al.^31^ proposed that the mechanisms of inhalation injury primarily involve the physical effects of heat, which directly damage the airway epithelium and, in severe cases, can affect the basal membrane, annular cartilage rings, and even the lung parenchyma, thereby altering the mechanical properties of the airway wall. Further investigation into these underlying mechanisms is essential to improve our understanding of heat-induced airway injury.

As shown in Fig. 5(b), the injury grading increases as the position approaches the ET tube, with slides from the high steam intensity level pig scoring higher and extending deeper into the carina compared with low and medium steam intensity level pigs. This is consistent with a picture that the steam produces greater injury at the ET tube where it is introduced, tapering off as it travels further toward the central airways and carina. This result is also consistent with the nCsC observations noted above. Associated ANOVA results (Table S4 in the Supplementary Material) reveal that the injury grading is indeed significant with position: the p-value for the position factor is very small ($5.82\times 10^{-8}$), indicating a significant effect of position on the injury grading. The linear regression model of injury grading with the steam intensity level and position (Table S5 in the Supplementary Material) also indicates a positive correlation, i.e., a more severe injury response close to the steam introduction site. Although histology remains the gold standard for identifying severe inhalation injuries (in tissue that can be harvested), it can be significantly influenced by the handling of the specimen during extraction and fixation. Conversely, aOCT is less affected by such artifacts and offers the additional benefit of capturing longitudinal data points as the injury progresses.

## 4 Conclusion

This study utilized an advanced endoscopic aOCT system integrated with pressure sensing capabilities to evaluate airway changes following steam inhalation injuries in a porcine model. Employing high-resolution aOCT imaging, the study quantified airway normalized nCsC after various steam injury intensity levels and at multiple post-injury time points. The histologic injury grading confirmed a higher severity of injury when closer to the steam introduction position (proximal end) and when more steam is introduced (higher steam intensity level). The nCsC results are consistent with the injury grading findings, which support our hypothesis that a decrease in nCsC is locally associated with an increase in injury severity. In addition, nCsC initially decreased in response to the injury and exhibited recovery after 4 h in cases of low and medium steam intensity levels. This provides insights beyond those offered by standard histology. Furthermore, aOCT also reveals histology-like details, for example, the thickening of the epithelial cell layer in the airway [Fig. 5(ii-b)], highlighting its potential for monitoring the progression of airway inhalation injuries.

The proposed aOCT elastography technology is promising toward clinical translation for assessing inhalation injury, with the hardware and procedures employed here ready to be used in humans with no salient differences. Future research should focus on specific diseases or injuries and monitor tissue changes over time, leveraging the minimally invasive nature of aOCT. A significant challenge encountered during this project was the breathing and cardiac movements of the test subjects. These movements can cause the aOCT catheter to contact the tissue surface, causing the scans to become unusable. The imbalance of usable data across different time points, positions, and steam intensity levels may reduce the overall statistical power. Moreover, it is challenging to keep the catheters centered within the airway to achieve optimal results. In addition, when recruiting subjects, it is crucial to consider the range of airway sizes; smaller airways increase the likelihood of the catheter contacting the airway wall, whereas larger airways may introduce an aliasing effect, although the latter was found to be recoverable with additional image processing. The translation from the pig study to pediatric studies is straightforward, as the size difference between pediatric and pig airways is minimal. For clinical studies involving adults, the protocol would need to be modified accordingly, and the aliasing correction method described in the Supplementary Material could be implemented.

In summary, our study not only advances the understanding of airway tissue responses to thermal injuries but also demonstrates the potential of aOCT elastography in providing valuable data that could be pivotal in clinical settings for assessing injury severity and monitoring recovery processes. This could eventually lead to more informed and targeted approaches to the management of inhalation injuries, optimizing therapeutic outcomes.

## 5 Appendix: Video Captions

The following video is mentioned in the text:

Video 1 Example of aOCT images (MP4, 8.91 MB [URL: https://doi.org/10.1117/1.JBO.30.7.076001.s1]).

## Supplementary Material

10.1117/1.JBO.30.7.076001.s01

10.1117/1.JBO.30.7.076001.s1

## Data Availability

Code and data can be made available upon request to the corresponding author.
