# Supplementary material for: In vivo assessment of airway wall compliance during inhalation injury response using anatomical optical coherence elastography
Source: J Biomed Opt. 2025 Jul 3;30(7):076001. doi: 10.1117/1.JBO.30.7.076001 (PMC12223793; doi:10.1117/1.JBO.30.7.076001)
Supplement: Supplementary file 1 [file JBO_030_076001_SD001.pdf]

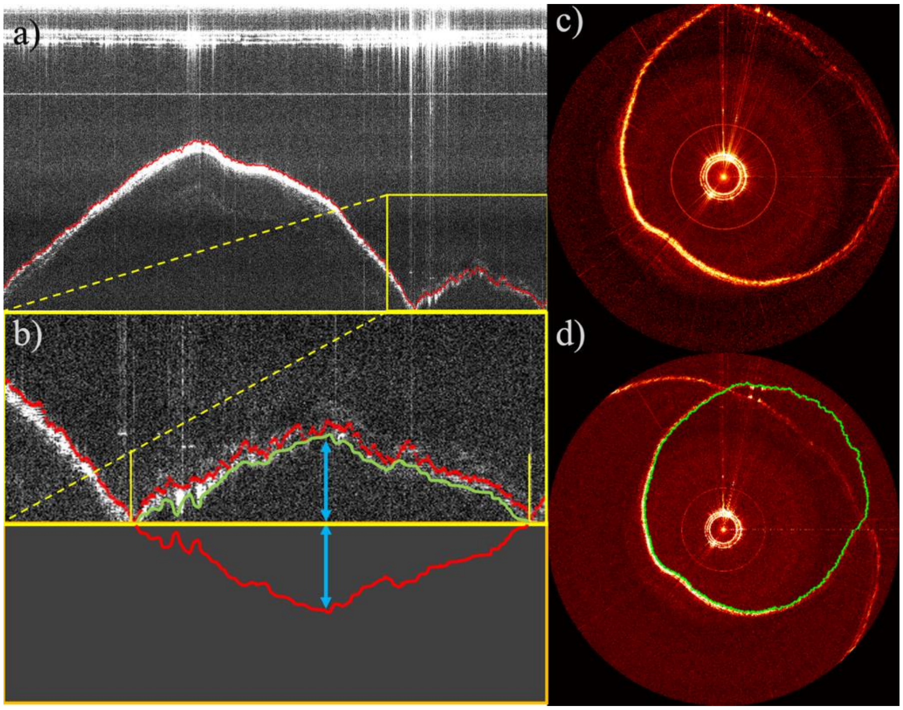

Figure S1. Aliasing segmentation correction. a) Rectangular aOCT image with initial segmentation line (red) overlayed. Yellow boxed region is the aliasing region. b) Zoomed region of yellow boxed area in a). Upper red dotted line in the upper panel in b) is the original segmentation line from semi-automated segmentation. Green line is the manually corrected inner surface of the airway. Lower red solid line is the mirrored (anti-aliased) segmentation of green line based on the bottom of the image. Two yellow vertical lines indicate the A-line where aliasing happened. Two blue arrows indicate the direction of the mirroring of the aliasing region segmentation. Lower panel in (b) is an added region to accommodate the mirrored segmentation. c) Original circular aOCT image with aliasing. d) Aliasing-corrected circular aOCT image with segmentation overlayed (green line).

Normalized Cross-sectional Compliance (nCsC):

If we assume the airway wall is an isotropic and linearly elastic material, the compliance can be described using a thick-walled tube model. The expressions of stress for the thick-wall tube model are:[32]

$$\sigma_r = \frac{A}{r^2} + B,\ \sigma_\theta = -\frac{A}{r^2} + B,\ \sigma_z = B, \quad (1)$$

where σ_r is the radial stress, σ_θ is the circumferential stress and σ_z is the axial stress, and

$$A = \frac{a^2 b^2 (p_o - p_i)}{b^2 - a^2},\ B = \frac{p_i a^2 - p_o b^2}{b^2 - a^2}, \quad (2)$$

where a is inner radius and b is outer radius, p_o is the outer pressure applied to the tube and p_i is the inner pressure applied to the tube.

Then the tangential strain is determined by:

$$\varepsilon_\theta = \frac{1}{E}(\sigma_\theta - \nu(\sigma_r + \sigma_z)), \quad (3)$$

where E is the Young's modulus and ν is the Poisson's ratio of the tube material.

If u is the radial displacement and v is the tangential displacement, then

$$\varepsilon_\theta = \frac{u}{v} + \frac{\partial v}{r \partial \theta}, \quad (4)$$

Because the system is axisymmetric, there is no angular dependence and the radial displacement can be written as $u = r\varepsilon_\theta$.

To model the change in cross-sectional area, we need to determine the change in radial (luminal) radius Δa, which can be calculated as the difference in u between two pressure values at the inner surface of the tube where $r = a$:

$$\Delta a = a(\varepsilon_\theta(r = a, p_i = p_o + \Delta p) - \varepsilon_\theta(r = a, p_i = p_o)), \quad (5)$$

Where $\Delta p = p_i - p_o$, then

$$\Delta a = a \cdot \Delta p \cdot x,\ x = \frac{1}{E}\frac{b^2(1+\nu) + a^2(1-2\nu)}{b^2 - a^2}, \quad (6)$$

Then the change in cross-sectional area due to the pressure change can be expressed as:

$$\Delta CSA = \pi(a + \Delta a)^2 - \pi a^2 \approx 2\pi a \Delta a, \tag{7}$$

where the approximation is valid when Δa is small compared to a. We then obtain for the compliance

$$CC = \frac{\Delta CSA}{\Delta p} \approx \frac{2\pi a^2 \Delta p x}{\Delta p} = 2\pi a^2 x. \tag{8}$$

The normalized cross-sectional compliance can then be expressed as:

$$nCsC = \frac{CC}{avgCSA} = \frac{2\pi a^2 x}{\pi a^2} = 2x. \tag{9}$$

Importantly, the normalization takes away much of the dependence on the overall cross-sectional size, with the caveat that the *x* parameter does still have a dependence on the inner and outer wall thicknesses (*a* and *b*, respectively). In the special case where *a*/*b* is constant (wall thickness scales with wall diameter), *x* is fully independent of size. Thus, the normalized cross-sectional compliance (nCsC) reflects the mechanical properties of the airway wall and is nominally independent of the cross-sectional area.

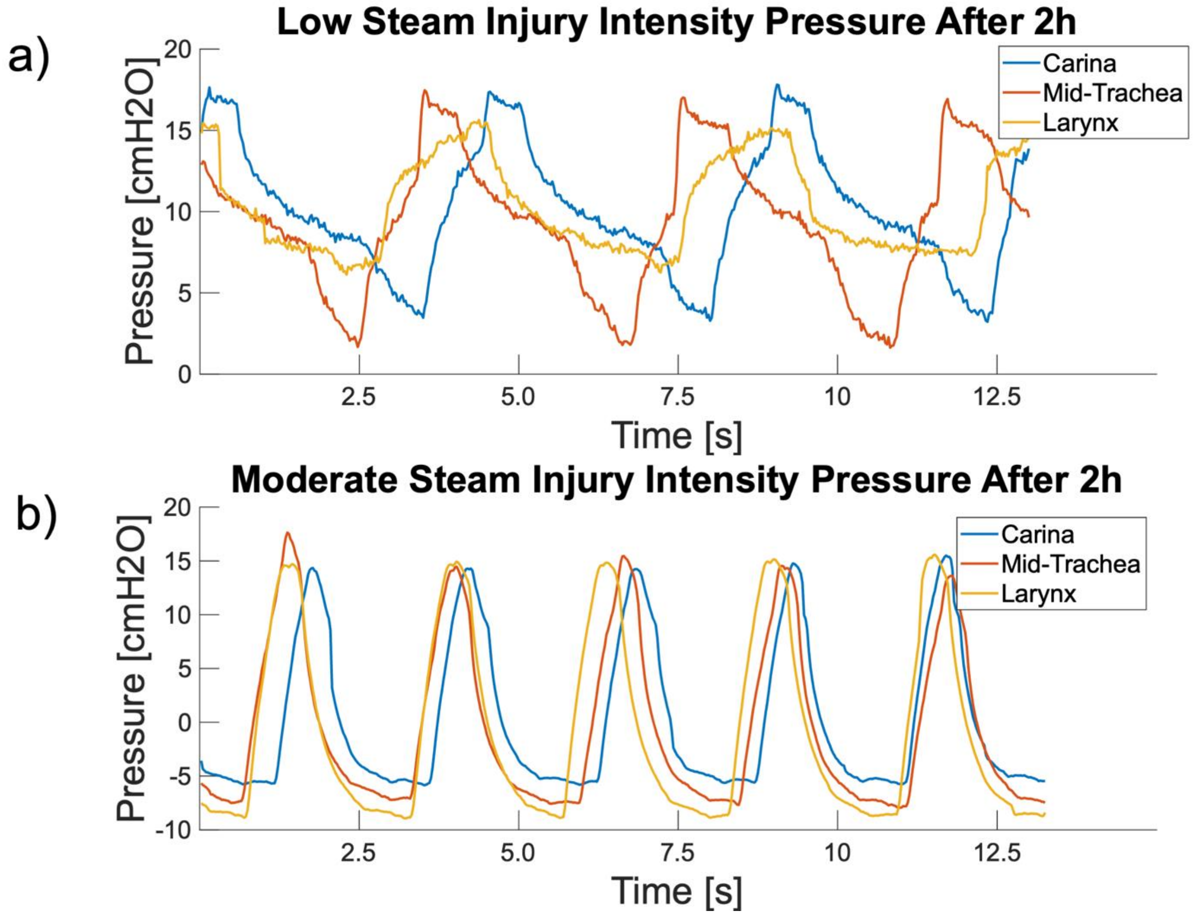

Figure S2. Pressure measured at different locations at the same time point on both low and moderate steam injury pigs. a) Low steam injury intensity pressure measurement at 2h after the injury at carina, mid-trachea and larynx. b) Moderate steam injury intensity pressure measurement at 2h after the injury at carina, mid-trachea and larynx.

Table S1. nCsC data collected from all available aOCT scans

Intensity Level	Time	Location*	Average CSA [mm^2]	CC [mm^2/cmH_2O]**	nCsC [$\%/cmH_2O$]**
Low	Before / 0h	0	79.2 ± 1.5	3.10 ± 0.43	3.92± 0.55
Low	Before / 0h	0.75	70.8 ± 1.0	1.13 ± 0.20	1.60 ± 0.29
Low	Before / 0h	0.85	70.7 ± 0.5	1.18 ± 0.18	1.63 ± 0.25
Low	Before / 0h	1	66.6± 2.2	1.12 ± 0.36	1.69 ± 0.55
Low	After / 0h	0.75	55.6 ± 1.4	0.56 ± 0.13	1.00 ± 0.24
Low	After / 0h	0.85	57.9 ± 0.2	0.54 ± 0.05	0.94± 0.08
Low	0.5h	0.66	58.3 ± 0.3	0.22 ± 0.04	0.39 ± 0.07
Low	0.5h	0.85	53.5 ± 0.5	0.84 ± 0.09	1.57 ± 0.17
Low	1h	0.85	53.0 ± 1.3	0.57 ± 0.09	1.07 ± 0.18
Low	2h	0	59.5 ± 0.9	0.57 ± 0.07	0.96 ± 0.12
Low	2h	0.66	60.1 ± 0.9	0.33 ± 0.06	0.55 ± 0.10
Low	2h	1	55.3± 0.4	0.80 ± 0.10	1.44 ± 0.18
Low	4h	0	65.2 ± 0.8	1.83 ± 0.32	2.81 ± 0.49
Low	4h	0.66	60.7 ± 0.8	0.72 ± 0.15	1.19 ± 0.25
Low	4h	0.75	58.5 ± 0.3	0.31 ± 0.07	0.54 ± 0.11
Low	4h	1	52.5 ± 0.4	1.19 ± 0.22	2.26 ± 0.43
Low	6h	0.66	55.5 ± 0.5	0.29 ± 0.07	0.52 ± 0.13
Low	6h	0.75	56.8 ± 0.4	0.23 ± 0.06	0.41 ± 0.10
Moderate	Before / 0h	0	118.4 ± 2.7	1.47 ± 0.28	1.24 ± 0.24
Moderate	Before / 0h	0.33	136.4 ± 3.2	3.99 ± 0.26	2.92 ± 0.19
Moderate	Before / 0h	1	131.5 ± 1.0	2.26 ± 0.13	1.72 ± 0.10
Moderate	2h	0	116.2 ± 1.2	0.90 ± 0.06	0.77± 0.05
Moderate	2h	0.33	106.1 ± 0.7	0.38 ± 0.03	0.36 ± 0.03
Moderate	2h	1	73.9 ± 0.4	0.27 ± 0.02	0.37 ± 0.02
Moderate	4h	0	116.4 ± 0.9	0.41 ± 0.03	0.36 ± 0.03
Moderate	4h	0.33	102.3± 0.9	0.30 ± 0.04	0.30 ± 0.03
Moderate	4h	1	69.4 ± 0.5	0.36 ± 0.02	0.52 ± 0.03
Severe	Before / 0h	0.66	82.6 ± 1.7	1.22 ± 0.18	1.52 ± 0.22
Severe	Before / 0h	1	81.2 ± 1.2	1.02 ± 0.19	1.25 ± 0.23
Severe	After / 0h	0.66	41.7 ± 0.8	0.38 ± 0.06	0.92 ± 0.14
Severe	After / 0h	1	60.2 ± 1.0	0.55 ± 0.10	0.92 ± 0.17
Severe	2h	0.33	61.4 ± 1.0	0.39 ± 0.06	0.63 ± 0.11
Severe	2h	0.66	56.9 ± 0.3	0.23 ± 0.02	0.41 ± 0.03
Severe	2h	1	43.6 ± 0.1	0.36 ± 0.02	0.83 ± 0.06

*The location column in the table uses a 0 to 1 system for labeling: 0 represents the distal end near the carina, and 1 represents the proximal end near the larynx, with decimal values indicating intermediate locations.
** Error bar means standard error.

Table S2. nCsC ANOVA results

Source	Df	Sum Sq	Mean Sq	F value	p value	
Intensity Level	1	1.271	1.271	2.659	0.115	
Position	1	0.650	0.650	1.360	0.254	
Time	1	5.506	5.506	11.515	0.002	**
Intensity Level : Position	1	1.637	1.637	3.424	0.076	.
Intensity Level : Time	1	0.588	0.588	1.230	0.278	
Position : Time	1	0.649	0.649	1.358	0.255	
Intensity Level : Position : Time	1	0.031	0.031	0.065	0.800	
Residual	26	12.432	0.478			
Total	33	22.764				

Signif. codes: 0 ‘***’ 0.001 ‘**’ 0.01 ‘*’ 0.05 ‘.’ 0.1 ‘ ’ 1
P values are Bonferroni corrected

Table S3. nCsC linear regression results

	Slope	Std. error	t-ratio	p value	
Intensity Level	-0.936	0.530	-1.768	0.089	.
Position	-2.473	1.186	-2.085	0.047	*
Time	-0.268	0.375	-0.716	0.481	
Intenstiy Level : Position	0.971	0.660	1.472	0.153	
Intensity Level : Time	-0.038	0.223	-0.172	0.865	
Position : Time	0.319	0.501	0.637	0.530	
Intensity Level : Position : Time	-0.076	0.296	-0.256	0.800	
Constant	3.558	0.932	3.817	0.001	

Signif. codes: 0 ‘***’ 0.001 ‘**’ 0.01 ‘*’ 0.05 ‘.’ 0.1 ‘ ’ 1
P values are Bonferroni corrected

Table S4. Histology score ANOVA results

	Df	Sum Sq	Mean Sq	F value	p value	
Intensity Level	1	0.404	0.404	1.391	0.738	
Position	1	14.926	14.926	51.463	5.82E-08	***
Intensity Level : Position	1	0.005	0.005	0.016	2.703	
Residual	36	10.441	0.290			
Total	39	25.776				

Signif. codes: 0 ‘***’ 0.001 ‘**’ 0.01 ‘*’ 0.05 ‘.’ 0.1 ‘ ’ 1
P values are Bonferroni corrected

Table S5. Histology score linear regression results

	Slope	Std. error	t-ratio	p value	
Intensity Level	0.128	0.226	0.568	1.720	
Position	2.209	0.736	3.001	0.015	*
Intensity Level : Position	-0.046	0.369	-0.125	2.703	
Constant	0.799	0.446	1.789	0.246	

Signif. codes: 0 ‘***’ 0.001 ‘**’ 0.01 ‘*’ 0.05 ‘.’ 0.1 ‘ ’ 1
P values are Bonferroni corrected
